# Pilot Study: Alteration of Deleted in Liver Cancer1 and Phosphorylated Focal Adhesion Kinase Y397 Cytoplasmic Expression and the Prognostic Value in Advanced Epithelial Ovarian Carcinoma

**DOI:** 10.3390/ijms12128489

**Published:** 2011-11-29

**Authors:** Dong-Mei Fan, Hui-Rong Shi

**Affiliations:** 1 Department of Obstetrics and Gynecology, The First Affiliated Hospital, Zhengzhou University, NO.1 Jianshe Road, Zhengzhou 450052, China; E-Mail: fandmhnhst103@sina.com; 2 Department of Gynecology, The First Affiliated Hospital of Henan University of Scientific and Technology, Luoyang 471003, China

**Keywords:** DLC1, gynecologic cancers, ovarian carcinoma, prognosis, p-FAK

## Abstract

**Background:**

Deletion in liver cancer gene (DLC1) and phosphorylated focal adhesion kinase (p-FAK) have recently been reported as metastasis-related genes. However, the roles and prognostic values of their expression in epithelial ovarian carcinomas (EOCs) remain unclear.

**Methods:**

The expression and prognostic value of DLC1 and p-FAK Y397 in EOC were evaluated by immunohistochemistry and multivariate analysis.

**Results:**

Low expression of DLC1 and high expression of p-FAK Y397 were found in the 76 cases of EOC. The expression of DLC1 and p-FAK Y397 were negatively correlated. Multivariate analysis showed that the combination of them was an independent prognostic marker of EOC (*P* = 0.0319).

**Conclusions:**

DLC1 and pFAK Y397 had an association with the clinicopathologic characteristics of EOC. Expression of neither of these genes was a prognostic factor alone, but the combination revealed a significant prognostic value in the 60 cases of advanced stage EOC.

## 1. Introduction

Ovarian carcinoma is the most lethal gynecological cancer in many countries [1]. Epithelial ovarian carcinomas (EOC) account for approximately 70% of all ovarian malignant diseases. Due to its insidious localization in the pelvis, almost 70% of EOC are found at an advanced stage (stage III/IV) [2]. The 5-year overall survival rate of patients with advanced stage cases is no more than 20%, compared to 90% for early stage cases (stage I/II) [3]. Although about 65–70% of the patients initially respond to the conventional treatment of cytoreduction, followed by a standard first-line chemotherapy regimen including combined platinum-paclitaxel, more than half of these patients relapse within a short period [4–6]. Because of the complex mechanism underlying the recurrence of ovarian carcinoma, many clinical indices such as FIGO stage, tumor type and the size of residual focus, do not always indicate a precise prognosis [4]. Therefore, developing a precise prognostic index would help us to not only understand the bona fide mechanism underlying EOC recurrence but also to construct a set of effective screening methods for the relapse of EOC [7]. Progress made toward increasing the survival rate of advanced stage EOC will improve the total survival rate of EOC.

Metastasis-related genes directly contribute to metastasis and recurrence, two factors that strongly influence the prognosis of cancer. The deletion in liver cancer gene (DLC1) is a tumor suppressor gene that was first identified by its deletion in a primary hepatocellular carcinoma sample [8] and subsequently found to be deleted or under-expressed in other cancers [9–13]. DLC1 is a GTPase-activating protein (GAP), and its deletion and resulting lack of interaction with a variety of downstream mediators causes tumorigenesis as well as uncontrolled cell growth [14,15]. Recently, abnormal, low, or lack of DLC1 expression was also associated with the metastasis of breast and hepatocellular cancers [16–18], suggesting that DLC1 plays an important role not only in tumorigenesis but also in metastasis.

Focal adhesion kinase (FAK) is an important oncogene that is upregulated in hepatocellular carcinoma and breast carcinoma [19,20]. DLC1 can inhibit the metastasis of hepatocellular carcinoma by dephosphorylation of FAK [16]. Abnormal expression of DLC1 and p-FAK in many cancers suggests that they are involved in the malignant behavior of tumors. p-FAK Y397 contains the major site of phosphorylation, tyrosine 397, makes FAK participated in the carcinogenesis of many cancers [16,21]. Yet, the roles of DLC1 and p-FAK Y397 as well as their potential prognostic values remain unknown for EOC. This study was undertaken to evaluate the expression of DLC1 and p-FAK Y397 in EOC by immunohistochemistry as well as to explore the roles of DLC1 and p-FAK Y397 in the prognosis of advanced stage EOC.

## 2. Materials and Methods

### 2.1. Patients

This study was undertaken at the First Affiliated Hospital of Zhengzhou University after approval from the Medical Ethical Committee was obtained. For the study, 76 patients who had undergone resection of EOC, 20 patients with borderline ovarian tumors and 20 patients with benign ovarian tumors were selected during 2000–2005. All of these patients underwent surgery at the Department of Gynecology, the First Affiliated Hospital of Zhengzhou University, between May 2000 and December 2005. Twenty cases of normal ovarian tissue came from patients with hysteroptosis who had their ovaries resected in transvaginal hysterectomy. All of the necessary informed consents were obtained. The average patients’ age at the time of surgery was 49 ± 16.6.

Years old (range: 30–71 years). None of the EOC patients received radio-chemotherapy or immunotherapy before surgery.

### 2.2. Sample Selection and Grouping

Paraffin-embedded tissues were obtained from patients with EOC (*n* = 76), borderline epithelial ovarian tumors (*n* = 20), benign epithelial ovarian tumors (*n* = 20) and normal ovarian tissue (*n* = 20). Immunohistochemical staining was used to detect the expression of DLC1 and p-FAK Y397. The explorative laparotomies, chemotherapies and treatment of patients included in this study were carried out at the First Affiliated Hospital of Zhegnzhou University. The mean follow-up was 36 months (range: 9–66 months). The patients with EOC stage I/II and in the borderline, benign, and normal groups were all alive at the end of follow-up. According to the FIGO 2000 staging standard, patients with EOC were classified after surgery as stage I/II (*n* = 16) and stage III/IV (*n* = 60). Surgery with residual tumor no larger than 1 cm was considered complete. Patients in stage III/IV who received 6 cycles of postoperative TC (135–175 mg/m^2^ paclitaxel and 300–400 mg/m^2^ carboplatin after calculating the concentration curve) with complete medical records (*n* = 60) were selected for overall survival. Clinicopathologic information was referred from medical records. All cases were diagnosed by two pathologists. The stage, grading, histology, age, familial history, malignant effusion and lymph metastasis of patients with EOC are summarized in Table 1.

### 2.3. Immunohistochemical Staining

Tissue samples were routinely fixed in 10% buffered formalin, processed and embedded in paraffin. Sections were cut at a thickness of 4 μm and dried overnight at 60 °C. After deparaffinization, the sections were rehydrated in graded dilutions of alcohol and incubated in 0.03% hydrogen peroxide for 10 min to block endogenous peroxidase. Sections were heated in a microwave oven to 98 °C for 15 min for antigen retrieval and washed in PBS three times. To avoid background staining, the sections were then pre-incubated with 5% w/v purified bovine serum albumin (BSA) (Dade Behring, Liederbach, Germany) diluted in Tris buffer pH 7.6 for 10 min. The sections were incubated with primary antibody overnight at 4 °C. The primary specific goat polyclonal antibody against human DLC1 (ab21200, Abcam) was diluted 1:50 (38 ng/mL) in 1% w/v purified BSA (Dade Behring) in Tris buffer pH 7.6, and the mouse monoclonal antibody against human p-FAK Y397 (sc-1688, Santa Cruz) was diluted 1:200 in the same solution. After incubation for 60 min, the sections were then incubated with streptavidin-peroxidase conjugate for 30 min at room temperature and washed with PBS (3 × 10 min). The color was developed with DAB added with chromogen for 10 min. Sections were rinsed in water, counterstained in Mayers Hematoxylin for 3 min and finally dehydrated, mounted and coverslipped with Pertex. Washings with 5 mM Tris buffer, pH 7.6 with NaCl 0.9% w/v and Tween 0.1% v/v (TBS) were used between all steps in the procedure. After heating, all steps were performed at room temperature in a humidity chamber to avoid air-drying of the sections. The positive control was a section from breast carcinoma. The negative control staining was done by omitting primary antibody.

Two pathologists who have experience evaluating immunohistochemically stained tissues immediately evaluated the expression of DLC1 and p-FAK Y397. A semi-quantitative scoring system was used to evaluate the IHC staining. The evaluation of IHC grade was performed by referring to the study of Hidemichi W [22]. A staining index was obtained from the multiplication of scores of the percentage of positive cells (0: <20% positive staining; 1: 20–80%; 2: >80%) and staining intensity (0: negative staining; 1: weak; 2: moderate; 3: strong). Scores varying from 0 to 6 were obtained by multiplying the two variables. Immunostaining of all slides was evaluated by specialists who were blind to the clinical data. Scores of 0–1 were defined as low expression (including negative staining), score 2 as moderate expression, and ≥3 as high expression. The staining of DLC1 and p-FAK Y397 were divided into negative groups (not stained at all) and positive groups.

### 2.4. Statistical Analysis

The association of expression of DLC1 and p-FAK Y397 in tumor patients was analyzed by Contingency Coefficient. Demographics data in DLC1 or p-FAK Y397 were compared using the Chi-square test. The expression rates of DLC1 or p-FAK Y397 in four groups were compared by logistic regression and Cochran-Armitage trend test. Overall survival was estimated by Kaplan-Meier curves. The log-rank test was used to compare the differences between survival curves. The Cox regression was used to calculate hazard ratios and 95% confidence intervals (CI). All of the statistical analyses were carried out with SAS version 6.12, and two-sided *P* < 0.05 was considered significant.

## 3. Results

### 3.1. Relationship Between the Expression of DLC1 and p-FAK Y397 in EOC

Among the 76 cases of EOC, there were 18 positive for DLC1 and negative for p-FAK Y397 as well as 41 negative for DLC1 and positive for p-FAK Y397. This result suggests a negative correlation between expression of DLC1 and p-FAK Y397 (by Contingency Coefficient *r* = −0.41, *P <* 0.001) (Table 1).

### 3.2. Expression of DLC1 and p-FAK Y397 in EOC

**Figure 1:**
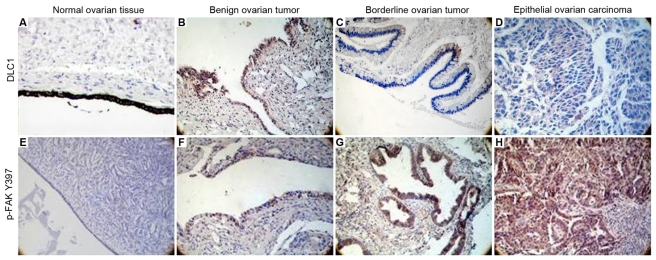
(**A–D**) Deleted in liver cancer 1 × 200 (DLC1) and (**E–H**) Phosphorylated focal adhesion kinase ×200 (p-FAK) Y397 protein expression in various ovarian tissues. Both DLC1 and p-FAK Y397 had yellow staining that was mainly localized to the cytoplasm. (**A**) DLC1 positive expression in normal ovarian tissue; (**B**) DLC1 positive expression in benign ovarian tumor; (**C**) DLC1 positive expression in borderline ovarian tumor; (**D**) DLC1 negative expression in epithelial ovarian carcinoma; (**E**) p-FAK Y397 negative expression in normal ovarian tissue; (**F**) p-FAK Y397 weak positive expression in benign ovarian tumor; (**G**) p-FAK Y397 positive expression in borderline ovarian tumor; (**H**) p-FAK Y397 positive expression in epithelial ovarian cancer.

**Figure 2:**
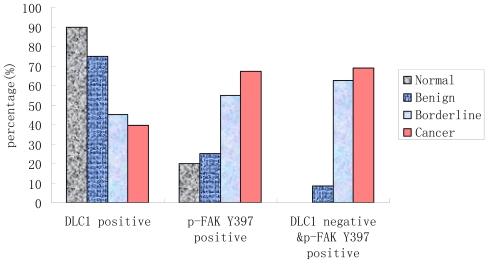
Bar graph for DLC1 and p-FAK Y397 expression in every group.

The positive immunostaining of DLC1 and p-FAK Y397 appears as cytoplasmic, granular brown-colored staining as shown in Figure 1. The frequencies of cytoplasmic expression of DLC1 in normal (90%), benign (75%) and borderline tissue (45%) were significantly higher than in EOC (39.47%) (*P* < 0.05, Figure 1A–D, Table 2). There was a decreased trend from the normal group to the EOC group (*Z*_trend_ = −4.47). However, the expression mode of p-FAK Y397 exhibited an opposite trend. The frequencies of cytoplasmic expression of p-FAK Y397 in normal (20%), benign (25%) and borderline tissue (55%) were significantly lower than in EOC (67.11%) (*P* < 0.05, Figure 1E–H, Table 2, Figure 2). There was an increased trend from the normal group to the EOC group (*Z*_trend_ = −4.50).

### 3.3. Relationship Between DLC1 or p-FAK Y397 Protein Expression and the Clinicopathological Parameters in 76 Cases of EOC

The expression of p-FAK Y397 in patients with lymph metastasis (80.49%) was significantly higher than in patients without lymph metastasis (51.43%) (*P* = 0.01). The expression of DLC1, however, showed no significant difference in lymph metastasis (*P* = 0.05). The expression of both DLC1 and p-FAK Y397 was associated with the stage (χ^2^ = 7.27, *P* < 0.05; χ^2^ = 5.01, *P* < 0.05) and ascite (*χ*^2^ = 16.73, *P* < 0.05; *χ*^2^ = 6.82, *P* < 0.05). The familial history, age, tumor histology and differentiation did not influence the expression of DLC1 and p-FAK Y397 (Tables 3 and 4).

### 3.4. Relationship Between DLC1 and p-FAK Y397 Protein Cytoplasmic Expression, Histological Differentiation and the Prognostic Factors of EOC

**Figure 3:**
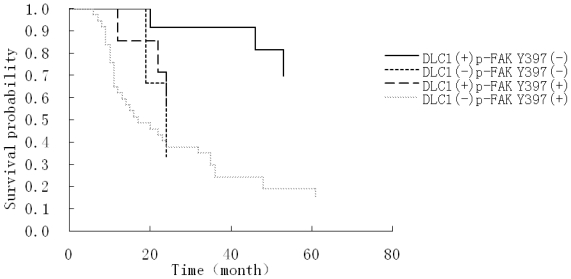
Kaplan-Meier cumulative survival curves showing the association between combining of DLC1 and p-FAK Y397 expression and overall survival.

**Figure 4:**
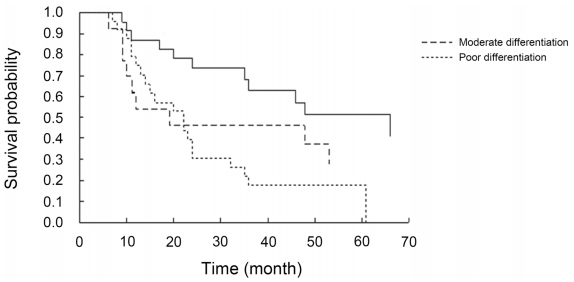
Kaplan-Meier cumulative survival curves showing the association between tumor grade of EOC and overall survival.

Univariate survival analysis demonstrated that histological differentiation, ascites, lymph metastasis, as well as expression of DLC1, p-FAK Y397 and DLC1 combined with p-FAK Y397 were significant correlative factors with prognosis. However, multivariate Cox analysis showed that only histological differentiation (*HR* = 2.467, 95% CI: 1.126–5.407) and DLC1 combined with p-FAK Y397 expression (*HR* = 3.922, 95% CI: 1.126–13.664) were independent risk factors that influenced the prognosis of advanced stage epithelial ovarian cancer (Table 5). In the 60 patients with advanced stage EOC, the overall survival of patients with tumors that stained positive for DLC1 and negative for p-FAK Y397 significantly differed from that of patients with tumors that stained negative for DLC1 and positive for p-FAK Y397 (*P* < 0.05). The mortality risk of the DLC1-negative and p-FAK Y397-positive patients was 3.922 times higher than for DLC1-positive and p-FAK Y397-negative patients. (Because the death number of the DLC1-positive and p-FAK Y397-negative patients was less than 50%, we used the mortality risk to represent the relationship between the DLC1, p-FAK Y397 and the overall survival status). Additionally, Kaplan-Meier survival curves confirmed that DLC1-negative and p-FAK Y397-positive patients in advanced stage had decreased overall survival time compared to the DLC1-positive and p-FAK Y397-negative patients (Figure 3). Similarly, the patients with well-differentiated tumors had a significantly longer overall survival than those with poorly differentiated tumors (median survival time 66 months versus 22 months). Patients with moderately differentiated tumors showed no difference in overall survival compared with patients who had poorly differentiated tumors (median survival time: 19 months and 22 months). Accordingly, Kaplan-Meier survival curves indicated that patients with well-differentiated tumors in advanced stage had increased overall survival compared to those with moderate and poor differentiation (Figure 4).

## 4. Discussion

DLC1 was recently identified due to its deletion in primary hepatocellular carcinoma and was mapped to 8p21.3–22, which is a reported site of tumor suppressor genes [9–13]. The gene structure of DLC1 consists of three main parts: sterile alpha motif (SAM), steroidogenic acute regulatory related lipid transfer domains (START) and Rho GTPase activating protein (RhoGAP). It is RhoGAP that makes DLC1 act as GTPase-activating protein (GAP). Rho family GTPases play important roles in the regulation of a variety of cellular processes including cell cycle progression, gene expression, cytoskeletal organization, cell morphology, cell migration, and cell adhesion to extra-cellular matrix [14]. DLC1 participates in the regulation of cytoskeletal rearrangement and morphological changes by inhibiting Rho GTPase activity [15]. For several kinds of cancers, DLC1 down-regulation or deletion was reported to leave the Rho/ROCK/MLC pathway and RhoA GTPase pathway more active [23,24], resulting in the cancer cells’ endless proliferation and migration.

In the present study, we found that the cytoplasmic expression of DLC1 protein was significantly decreased or absent in EOC when compared with other types of ovarian tumor tissues and normal ovarian tissues. Previous studies have assessed the expression of DLC1 mRNA in EOC [25]. According to Dietmar’s RT-PCR data, DLC1 mRNA was significantly down-regulated in EOC [25], but the abnormal expression of DLC1 mRNA was not related to the clinicopathological parameters of EOC. The present study, however, indicated that the decreased expression of DLC1 protein was related with the FIGO stage and malignant effusions. Based on Dietmar’s study, our data suggest that post-transcriptional control of DLC1 might affect the development of EOC. Metastasis is a direct factor in the recurrence of many cancers and an immediate index of prognosis of cancers [26–28]. Micrometastatic lesions and circulating tumor cells [29,30] might contribute to the relapse of the cancer. Therefore, metastasis-related genes play a key role in predicting the prognosis of cancer. In breast cancer, DLC1 was shown to be down-regulated by thrombospondin2 (TSP2) and was implicated as a contributor to the micrometastasis of breast cancer [21]. In a study by Zhang *et al*., expression of DLC1 was intimately correlated with the metastasis and prognosis of clear cell renal cell carcinoma [31]. In concordance with these studies, our data indicate that deactivation of DLC1 was an importment molecular changement in EOC.

FAK is a non-receptor tyrosine kinase. As such, FAK can be activated at tyr397 by many protein tyrosine kinases such as PI3K and Grb7 and subsequently regulate focal adhesion dynamics during cell migration and activate prosurvival and mitogenic signaling pathways [20,21]. Published studies have reported high expression of p-FAK in cancers like hepatocellular carcinoma [19] and breast carcinoma [20]. Our data indicate that the cytoplasmic expression of p-FAK Y397 was higher in EOC than other types of ovarian tissues. Moreover, its high expression was closely related to the FIGO stage, malignant effusions and metastastic lymph nodes of EOC. Consistent with the study in breast carcinoma [20], the results of this study suggest that the phosphorylation of FAK might be another molecular changement closely related with EOC.

The difference of correlation with lymph metastasis between DLC1 and p-FAK Y397 might be due to p-FAK’s central involvement in many signaling pathways. The complex integration of many pathways might contribute to p-FAK’s involvement in the lymph metastasis of EOC. By acting on various downstream molecules, DLC1 controls cell growth and migration in different kinds of cancers [16,32–34]. As one of the downstream genes of DLC1, FAK can be dephosphorylated on tyr397 and tyr925; the subsequently down-regulated p-FAK Y397 and p-FAK Y925 are able to play important roles in tumor suppression and metastasis suppression [16]. It is worth noting that we have discovered an obvious negative correlation between the expression of DLC1 and p-FAK Y397 in EOC: lower expression of DLC1 was associated with higher expression of p-FAK Y397 in EOC. This result indicates that abnormal DLC1 expression might contribute to EOC by altering the expression of p-FAK Y397. Kim *et al*. found that after transfection of DLC1 in hepatocellular carcinoma cells, the expression of p-FAK, paxillin and p130cas were down-regulated and cell proliferation and migration were inhibited [16]. Our results, combined with other studies, indicated that DLC1 and p-FAK Y397 might be promising new therapeutic targets for EOC, though their value for this application requires further verification. We also found that cytoplasmic expression of DLC1 and p-FAK Y397 did not correlate with familial history, suggesting DLC1 and p-FAK Y397 are not factors in heredity EOC.

Differentiation has always been demonstrated as an independent prognostic factor of EOC [35–37]. Cho *et al*. [35] held that the differentiation of EOC determined the cell biology and the subsequent response to therapy, making differentiation an independent prognostic factor of EOC not only in stage I but also in advanced stage. The data from the present study also support these conclusions.

Although the univariate analyses indicated that both DLC1 and p-FAK were prognostic factors, on multivariate Cox regression analysis they were found to not be independent risk factors for the prognosis of EOC in advanced stage. Notably, the combination of DLC1 and p-FAK Y397 was an independent risk factor of EOC in advanced stage, and this was confirmed by multivariate Cox regression analysis. The overall survival time for the DLC1-negative and p-FAK Y397-positive group was short, while that for the DLC1-positive and p-FAK Y397-negative group was relatively long. Log-rank test revealed significant differences between the two groups. A similar trend was observed in the well-differentiated and moderate- to poorly-differentiated groups. The survival analysis data in the present study strengthens the notion that DLC1 contributes to the carcinogenesis and metastasis of advanced stage EOC by interacting with FAK. Table 4 shows that there was no association between combining of DLC1 and p-FAK Y397 expressions and tumor differentiation (*P* = 0.7038). On one hand, this data just supports the idea that both combining of DLC1 and p-FAK Y397 expressions and differentiation was the independent prognosis factor for EOC. On the other hand, this means that the alteration of DLC1 and p-FAK Y397 might not be the intrinsic mechanisms for the differentiation of EOC. Besides metastasis, chemoresistance can also directly cause relapse and impact the prognosis of EOC. Studies have found that p-FAK might be involved in the chemoresistance of EOC and other cancers [38,39]. The significant prognostic value of p-FAK Y397 also hints that therapy response might be another way that p-FAK directly correlates with the prognosis of advanced stage EOC, though this requires further confirmation. It seems that in this pilot study, histological differentiation remains a more accurate independent prognostic factor (*P* = 0.0240) than DLC1 and p-FAK (*P* = 0.0319).

The presented data showed that DLC1 combined with p-FAK Y397 was an independent risk factor of advanced stage EOC as well as histological differentiation. Targeting either DLC1 or p-FAK Y397 might be a potential strategy for controlling the metastasis of EOC.

## Figures and Tables

**Table 1:** Relationship between the expression of DLC1 and p-FAK Y397 in EOC.

	p-FAK Y397(−)	p-FAK Y397(+)	χ^2^	*P*
DLC1(−)	8	38	12.6889	<0.001
DLC1(+)	17	13		

**Table 2:** DLC1 and p-FAK Y397 expression in various kinds of epithelial ovarian tissues.

Group	DLC1 Positive Rate (%)	OR (95% Confidence Interval)	p-FAK Y397 Positive Rate (%)	OR (95% CI)	DLC1 Negative & p-FAK Y397 Positive *n* (%)	OR (95% CI)
Normal	18 (90.00)		4 (20.00)		0 (0.00)	
Benign	15 (75.00)	0.333 (0.056–1.971)	5 (25.00)	1.333 (0.300–5.925)	1 (8.33)	-
Borderline	9 (45.00)	0.091 (0.017–0.501)	11 (55.00)	4.889 (1.199–19.942)	5 (62.50)	-
Cancer	30 (39.47)	0.072 (0.016–0.335)	51 (67.11)	8.16 (2.469–26.972)	38 (69.09)	-

Compared to the normal group, the positive expression of DLC1 in the borderline and EOC groups were significantly low (Trend test, *Z* = −4.47, *P* < 0.001); p-FAK Y397, however, showed a negative trend (Trend test, *Z* = −4.50, *P* < 0.001); “-”: not computed; *n*: number.

**Table 3:** Clinical characteristics and expression of DLC1 and p-FAK Y397 in epithelial ovarian carcinoma.

		DLC1 (*n*)				p-FAK Y397 (*n*)			
		
		(−)	(+)	χ^2^	*P*	(−)	(+)	χ^2^	*P*
Age	≤50	19	13	0.03	0.86	10	22	0.07	0.79
	>50	27	17			15	29		
FIGO stage	I~II	5	11	7.27	0.01 *	9	7	5.01	0.03 *
	III~IV	41	19			16	44		
Lymph metastasis	N	17	18	3.88	0.05	17	18	7.22	0.01 *
	Y	29	12			8	33		
Ascites	N	8	19	16.73	<0.001 **	14	13	6.82	0.01 *
	Y	38	11			11	38		
Histological differentiation	poor	14	12	1.22	0.54	8	18	0.99	0.61
	moderate	11	8			8	11		
	well	21	10			9	22		
Familial history	Y	44	27	0.94	0.34	23	48	0.12	0.73
	N	2	3			2	3		

* *P* < 0.05;

** *P* < 0.001.

**Table 4:** The association between combining of DLC1 and p-FAK Y397 expressions and tumor differentiation.

	Poor	Moderate	Well	χ^2^	*P*
DLC1(+) & p-FAK Y397(−)	7(41.18)	4(23.53)	6(35.29)	0.7024	0.7038
DLC1(−) & p-FAK Y397(+)	13(34.21)	7(18.42)	18(47.37)		

**Table 5:** Prognostic analysis of 60 cases of epithelial ovarian carcinoma.

	Parameter Estimation	Standard Error	χ^2^	*P* Value	Hazard Ratio (*HR*)	95% Confidence Interval	
Histological differentiation moderate *vs.* well	0.75737	0.46074	2.7021	0.1002	2.133	0.864	5.261
Histological differentiation poor *vs.* well	0.90318	0.40025	5.0919	0.0240	2.467	1.126	5.407
Lymph metastasis	0.24549	0.41800	0.3449	0.5570	1.278	0.563	2.900
Ascites	1.07839	0.65236	2.7326	0.0983	2.940	0.819	10.559
DLC1	0.20422	0.53087	0.1480	0.7005	1.227	0.433	3.472
p-FAK Y397	1.02819	0.59392	2.9971	0.0834	2.796	0.873	8.955
DLC1 combined with p-FAK Y397	1.36657	0.63686	4.6045	0.0319	3.922	1.126	13.664
